# Carbon dioxide and MAPK signalling: towards therapy for inflammation

**DOI:** 10.1186/s12964-023-01306-x

**Published:** 2023-10-10

**Authors:** Hanna Gałgańska, Wieslawa Jarmuszkiewicz, Łukasz Gałgański

**Affiliations:** 1 Faculty of Biology, Molecular Biology Techniques Laboratory, Adam Mickiewicz University in Poznan, Uniwersytetu Poznanskiego 6, 61-614 Poznan, Poland; 2 Faculty of Biology, Department of Bioenergetics, Adam Mickiewicz University in Poznan, Institute of Molecular Biology and Biotechnology, Uniwersytetu Poznanskiego 6, 61-614 Poznan, Poland

**Keywords:** ERK1/2, p38, JNK, Hypercapnia, Hypercarbia, Hypocapnia

## Abstract

Inflammation, although necessary to fight infections, becomes a threat when it exceeds the capability of the immune system to control it. In addition, inflammation is a cause and/or symptom of many different disorders, including metabolic, neurodegenerative, autoimmune and cardiovascular diseases. Comorbidities and advanced age are typical predictors of more severe cases of seasonal viral infection, with COVID-19 a clear example. The primary importance of mitogen-activated protein kinases (MAPKs) in the course of COVID-19 is evident in the mechanisms by which cells are infected with SARS-CoV-2; the cytokine storm that profoundly worsens a patient’s condition; the pathogenesis of diseases, such as diabetes, obesity, and hypertension, that contribute to a worsened prognosis; and post-COVID-19 complications, such as brain fog and thrombosis. An increasing number of reports have revealed that MAPKs are regulated by carbon dioxide (CO_2_); hence, we reviewed the literature to identify associations between CO_2_ and MAPKs and possible therapeutic benefits resulting from the elevation of CO_2_ levels. CO_2_ regulates key processes leading to and resulting from inflammation, and the therapeutic effects of CO_2_ (or bicarbonate, HCO_3_^−^) have been documented in all of the abovementioned comorbidities and complications of COVID-19 in which MAPKs play roles. The overlapping MAPK and CO_2_ signalling pathways in the contexts of allergy, apoptosis and cell survival, pulmonary oedema (alveolar fluid resorption), and mechanical ventilation–induced responses in lungs and related to mitochondria are also discussed.

Video Abstract

**Supplementary Information:**

The online version contains supplementary material available at 10.1186/s12964-023-01306-x.

## Introduction

The SARS-CoV-2 pandemic highlighted how insufficiently we clinically treat excessive inflammation. Although the specific mechanisms leading to the production of proinflammatory cytokines and the activation of immune system components, as well as the signalling and other effects of proinflammatory cytokines and chemokines, are relatively well understood, the interrelationships among these factors are largely unclear. Due to the many mechanisms common to various pathologies and pathogen infections, including signalling pathways leading to inflammation and activated in response to inflammation, it is worth evaluating lessons learned during the COVID-19 pandemic, making conclusions about the treatments provided, and continuing the intensive search for effective therapies for inflammation.

Mitogen-activated protein kinases (MAPKs) regulate cell proliferation, survival, differentiation, migration, and apoptosis; oncogenesis; and neurodegeneration [1–5]. Signals from cellular receptors are transduced by MAPKs to a wide variety of effector proteins, including transcription factors, which regulate cell functions according to environmental conditions. In this review, we focus on three subfamilies of MAPKs, namely, c-Jun N-terminal kinases (JNKs), extracellular signal-regulated kinases 1 and 2 (ERK1/2) and p38 MAPKs, as they are key players in the regulation of inflammation and play important roles in signalling pathways critical to the course of SARS-CoV-2 infection. In response to a wide variety of chemical and biological agents, these MAPKs not only promote the production of reactive oxygen species (ROS) and proinflammatory cytokines, including interferon-gamma (IFN-γ), interleukin-1β (IL-1β), interleukin-6 (IL-6) and tumour necrosis factor-α (TNF-α) but also regulate cellular responses to a wide range of cytokines [6–9].

**Fig. 1:**
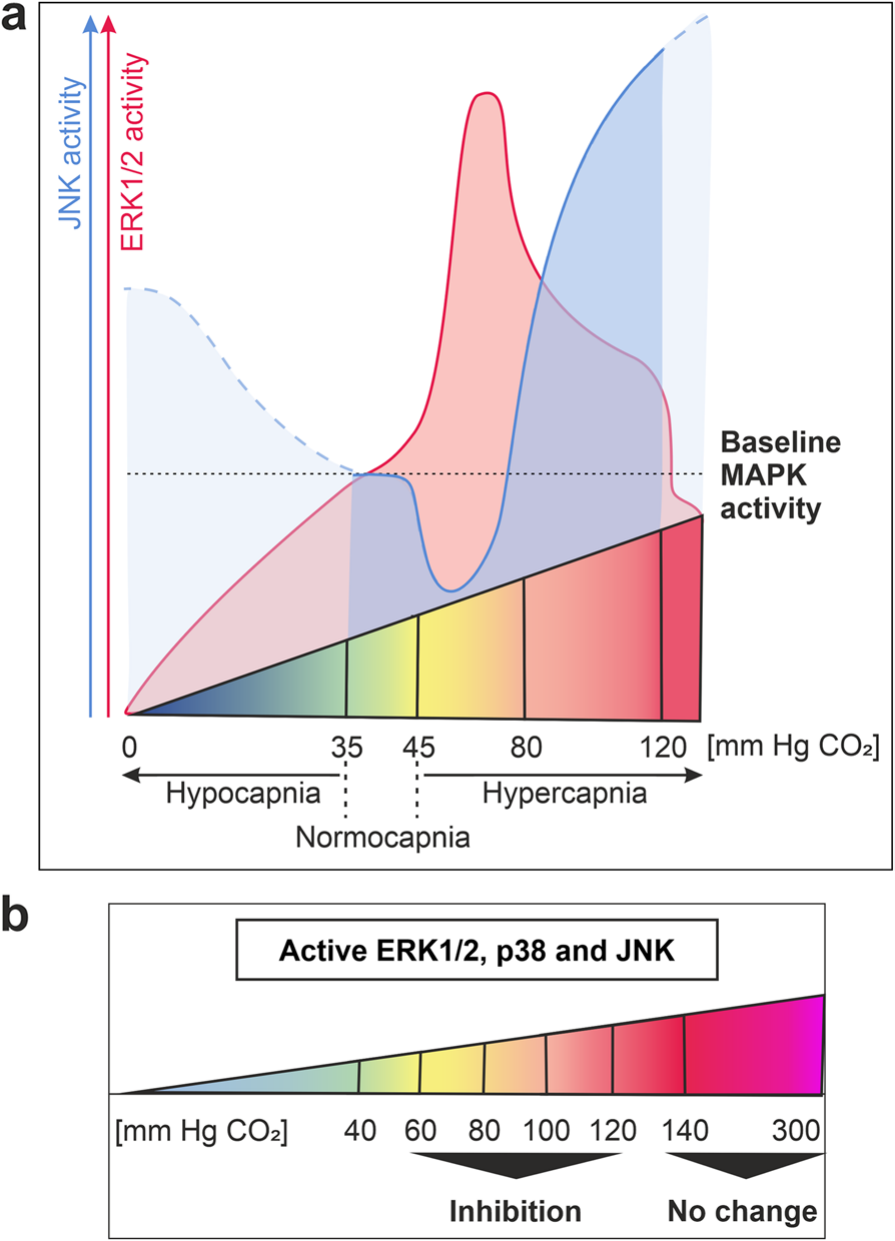
Profiles of MAPK activity in response to elevated CO_2_ based on the data shown in Table 1. **a** CO_2_-dependent regulation of baseline ERK1/2 and JNK levels. The blue dashed line represents the extrapolated JNK activity values. **b** Inactivation of active MAPKs by CO_2_ at elevated levels

**Fig. 2:**
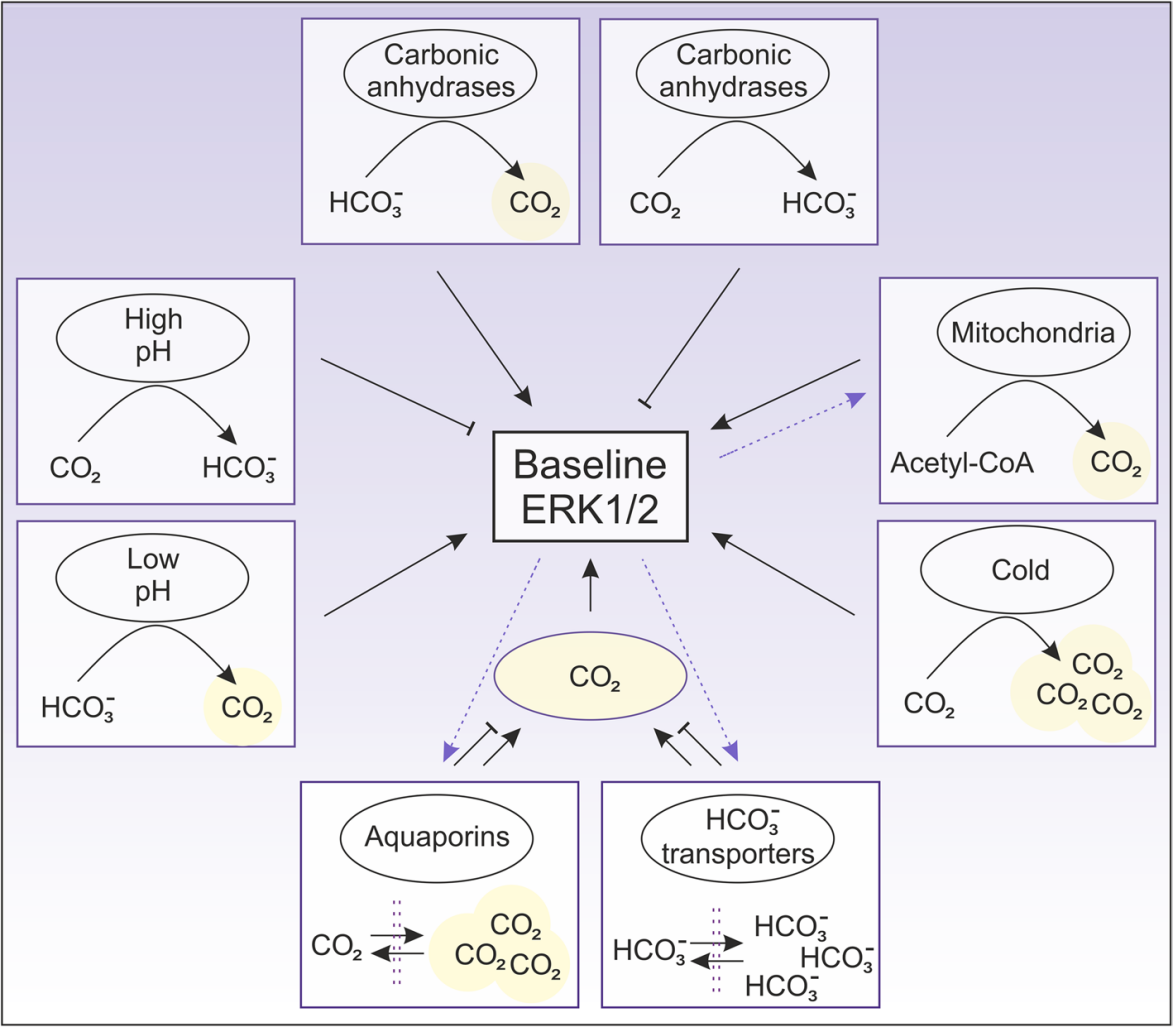
Cellular mechanisms regulating CO_2_-dependent activation of ERK1/2

MAPKs have been proposed to be carbon dioxide (CO_2_) sensors because select MAPKs have been shown to be highly regulated by CO_2_ in vitro, in human cells and in plants [10–13]. CO_2_-dependent regulation of MAPKs has been demonstrated in several animal cell types and tissues (Table 1), but the direct influence of CO_2_ on MAPK activity has not been previously considered. The effects of CO_2_ on MAPK activity are very dynamic and depend on the concentration of CO_2_ and the duration of CO_2_ exposure. Although the mechanisms of action underlying the effect of CO_2_ on MAPK functions remain unclear, an emerging pattern indicates that inactive ERK1/2 and plant ERK-type MAPKs are activated, and the functions of all the activated MAPKs studied thus far have been inhibited by increased CO_2_ levels (Figs. 1 and 2). Importantly, among several groups of proteins proposed to be CO_2_ sensors, only MAPKs are common to all eukaryotes, with the other eukaryotic CO_2_ sensors being taxon specific.

**Table 1:** Signalling pathways with MAPK activity regulated by CO_2_

MAPKs	CO_2_ levels	Duration of CO_2_ treatment	Starting MAPK activity	Change in MAPK activity in response to CO_2_	Signalling pathway	Cell/tissue type	Ref
ERK1/2	6.5–15%	2–15 min	Baseline	↑	CO_2_ signalling	Cultured endothelial cells and bronchial epithelial cells	[10]
ERK1/2	10%	5–15 min	Baseline	↑	Cell proliferation	Human small cell lung cancer cell line	[14]
ERK1/2	120 mm Hg	5–30 min	Baseline	↑	CO_2_ signalling	A549 cells	[11]
ERK1/2	120 mm Hg	1–30 min	Baseline	↑	CO_2_ signalling	Rat ATII cells	[12]
ERK1/2	20% (120 mm Hg)	2 h	Baseline	↑	CO_2_ signalling	PC-12 rat pheochromocytoma cells	[15]
ERK1/2	hypocapnic 1000–1200 ppm	30 min	Baseline	↓	CO_2_ signalling	Rat hindleg skeletal muscle, ex vivo	[16]
ERK1/2	40–45% CO_2_ (∼300 mm Hg)	25 min	Baseline	No change	CO_2_ signalling	Rat cingulate cortex	[17]
ERK1/2, p38, JNK	20% (140 mm Hg)	5–60 min	Active	No change	Innate immune responses to LPS	PMA-differentiated THP-1 macrophages	[18]
ERK1/2	6.5–13%	7–15 min	Active	↓	H_2_O_2_, proinflammatory cytokines, SARS-CoV-2 spike protein	Cultured endothelial cells and bronchial epithelial cells	[10]
ERK1/2	percutaneous 100% CO_2_ mist	10 min once a day	Active	↓	Ischaemia	Rodent hindlimb muscle	[19]
ERK1/2	12%	1 h in vitro, 3 h in vivo	Active	↓	Ventilator‐induced lung injury, cyclic stretch	Murine lung, rat primary AECs	[20]
ERK1/2, p38	10%	Overnight	Active	↓	Insulin resistance in post-surgical trauma	Adipocytes	[21]
ERK1/2, JNK	100%	5 min	Baseline	↓	Euthanasia – CO_2_ asphyxiation	Murine brain	[22]
p38	100%	5 min	Baseline	No change	Euthanasia – CO_2_ asphyxiation	Murine brain	[22]
p38	> 60 mm Hg	1 h	Active	↓	Retinal ischaemia–reperfusion injury	Retinal neural cell line	[23]
p38, JNK	8%	2–15 min	Baseline	↓	CO_2_ signalling	Cultured endothelial cells	[10]
p38, JNK	80–100 mm Hg	4 h	Active	↓	Injury ventilation; high-pressure mechanical stretch	Rat lungs (primary ATII cells)	[24]
JNK	80–120 mm Hg	10–15 min	Baseline	↑	CO_2_ signalling	ATII cells	[25, 26]
JNK	120 mm Hg	1–15 min	Baseline	↑	CO_2_ signalling	A549 cells	[11]
JNK	60 mm Hg	1–15 min	Baseline	No change	CO_2_ signalling	ATII cells	[26]

**Fig. 3:**
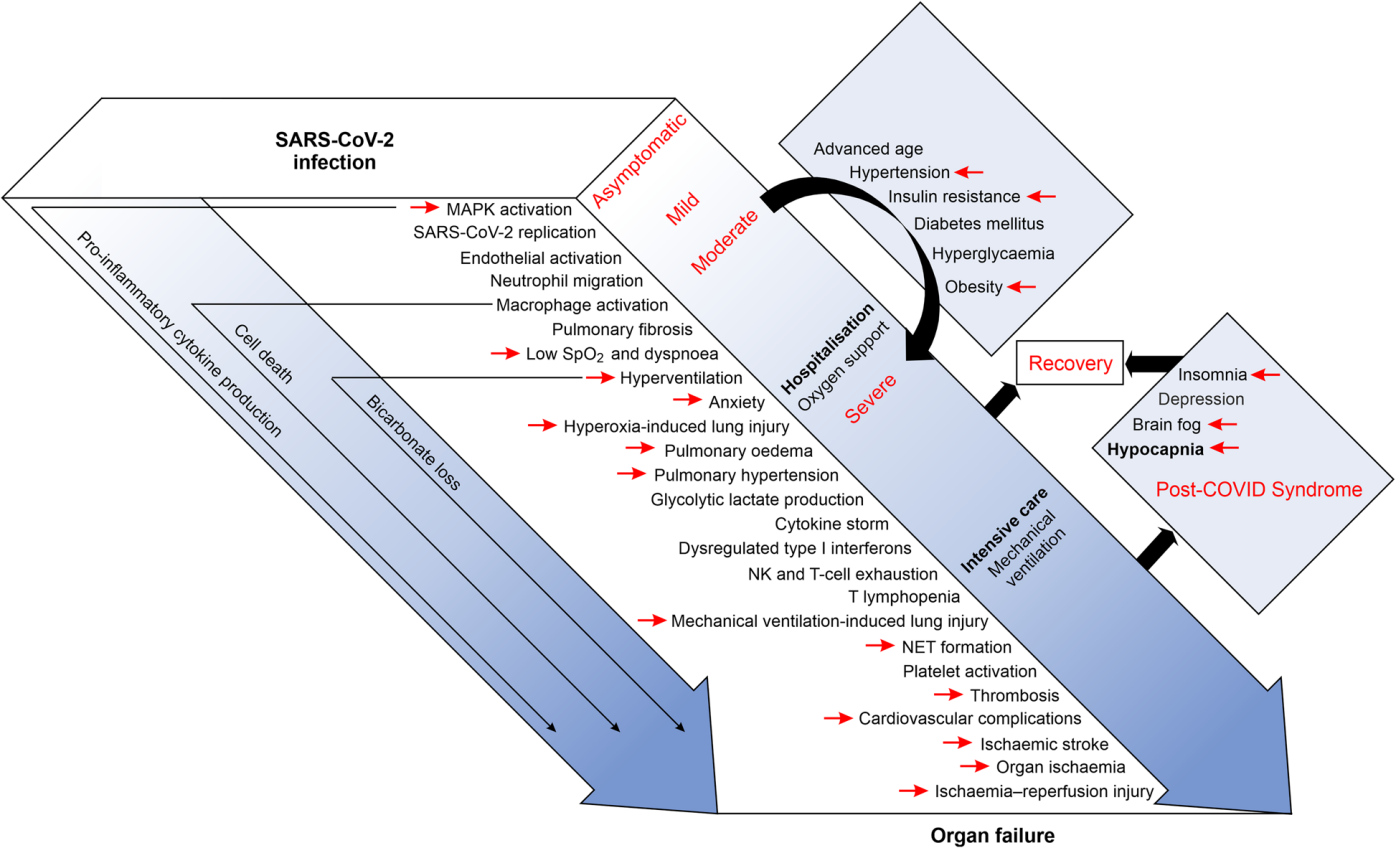
An overview of the involvement of MAPKs and the potential beneficial effects of elevated CO_2_ levels on the pathogenesis of COVID-19 and comorbidities. Red arrows: confirmed impact of elevated CO_2_ levels

**Fig. 4:**
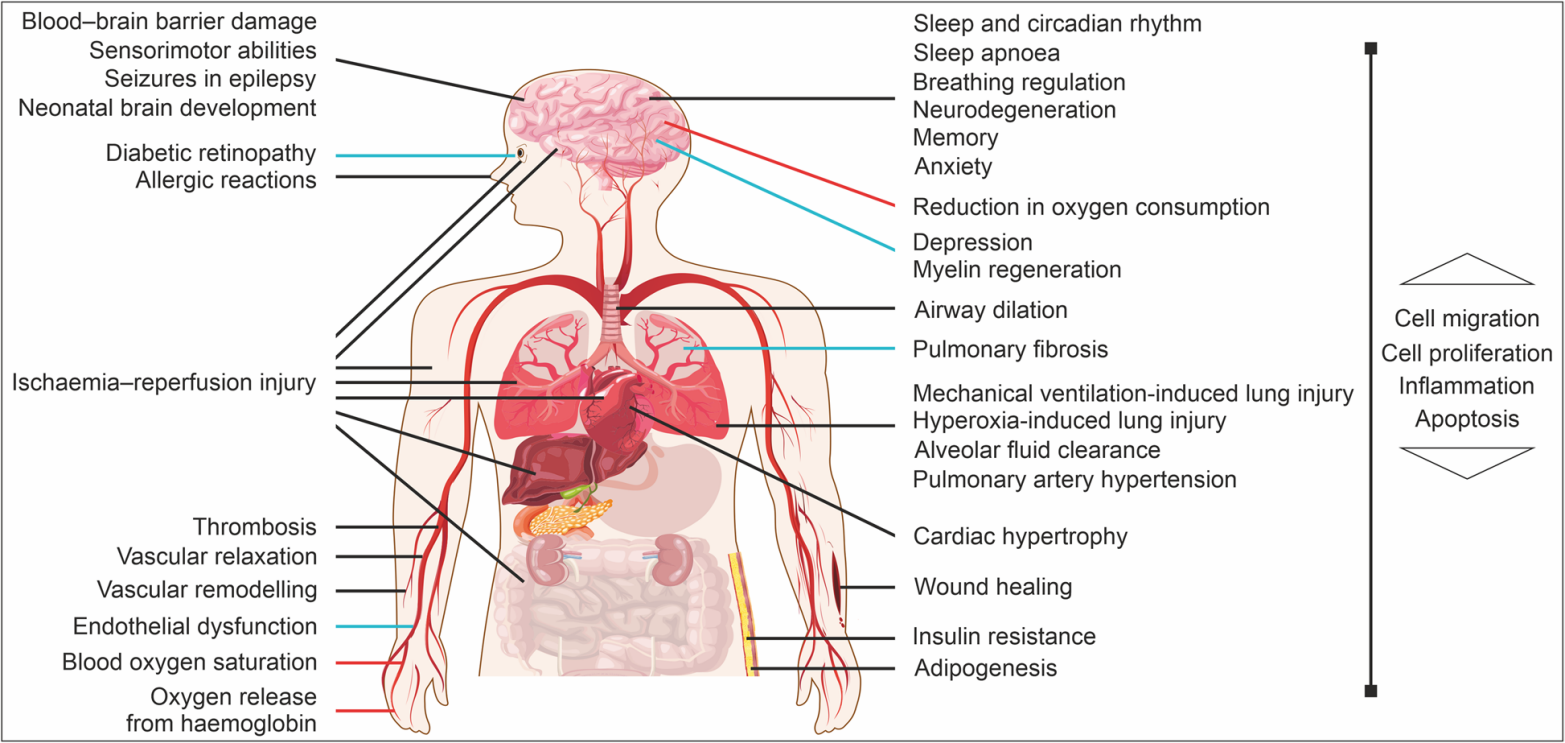
The physiological and pathological processes regulated by MAPKs and/or CO_2_. Blue lines: mechanisms regulated by MAPKs; red lines: influence of elevated CO_2_ levels; black lines: overlapping effects of MAPKs and CO_2_

In the following parts of this paper, we highlight ERK1/2-dependent processes that are augmented by an increase in CO_2_ concentration and the harmful effects triggered by MAPK signalling that can be inhibited by elevating CO_2_ levels. We indicate possible or previously observed consequences of purposely increasing CO_2_ levels in relation to various aspects of COVID-19 and the most common comorbidities in patients with COVID-19. Since the pathogenesis and therapy of COVID-19 is an extremely broad topic, including the roles of MAPKs and CO_2_ in these contexts, we do not describe all the possible benefits of increasing CO_2_ levels in COVID-19 therapy in this article, as these benefits have been discussed in other recently published papers [27–30]. We focus primarily on the cooperation of the CO_2_–MAPK signalling module, because the functions of CO_2_ and MAPK largely overlap (Table 2, Figs. 3 and 4), and it has recently been suggested that MAPKs may be CO_2_ receptors [10]. Due to text length limitations, we mainly emphasize the benefits of a transient increase in CO_2_ levels. A broader view of hypercapnia can be found in many recent review papers [31–33].

**Table 2:** Signalling pathways with overlapping effects of MAPK activity and elevated CO_2_ levels

Process	MAPKs	CO_2_ intervention	Identified mechanisms	References	
				CO_2_	MAPK
Alveolar fluid resorption	ERK1/2, p38, JNK	10% CO_2_ for 20 min in human AECs; 5–12% CO_2_ in rodent models	Regulation of ion and water flow by ENaC, Na/K-ATPase, CFTR and AQPs, regulation of intracellular cAMP levels	[31, 34–37]	[11, 12, 25, 26, 38–47]
LPS-induced lung injury	ERK1/2	2.5–20% CO_2_ (lung macrophages), prophylactic or therapeutic 5% CO_2_ inhalation	Cytokine responses in alveolar macrophages, downregulation of Toll-like receptor 4 expression, NF-κB signalling	[36, 48, 49]	[50–52]
Mechanical ventilation-induced lung injury	ERK1/2, p38, JNK	12–15% CO_2_ (AECs), 80–100 mm Hg PaCO_2_ (ventilated rats)	Regulation of NF-κb, ICAM-1, ADAM17, IL-6, IL-8, epidermal growth factor receptor (EGFR) activity, lung infiltration by neutrophils and AEC apoptosis	[20, 24, 53–55]	[20, 24, 56]
Hyperoxia	ERK1/2, p38, JNK	Immersion of lower legs in CO_2_-enriched (1,553 mg CO_2_/l) water or 60–146 mm Hg PCO_2_ (cells, bioptates or the organism)	Hyperoxia-induced cell apoptosis; NADPH-oxidase activity; production of O_2_^.^, antioxidants and proinflammatory cytokines; Nrf2, adenosine A2A receptor, protein kinase A (PKA), Src, cAMP, small mothers against decapentaplegic 3 (SMAD3), semaphorin 3A and A-kinase anchoring protein 1 (Akap1) signalling pathways	[57, 58]	[59–66]
Airway dilation	ERK1/2, p38	Increased CO_2_ concentrations in the bath (isolated bronchial rings), an increase in EtCO_2_ of 1 kPa (healthy volunteers and asthma patients), inhaled 5–10% CO_2_	Akt-C/EBPβ-CCL20-mediated epithelial-mesenchymal transition; NLRP3 deubiquitination and transcriptional upregulation leading to NLRP3 inflammasome activation, voltage-dependent Ca^2+^ channels; Ca^2+^ and substance P signalling	[67–74]	[75, 76]
Pulmonary artery hypertension	ERK1/2, p38	EtCO_2_/PaCO_2_ measurement in patients with PAH, 5% CO_2_ for 10 min (isolated perfused rat lungs)	15-Hydroxyeicosatetraenoic acid (15-HETE) and 15-lipoxygenase-2 signalling, mitosis and apoptosis of pulmonary arterial smooth muscle cells, differentiation of mesenchymal stem cells leading to vascular remodelling	[77–79]	[80, 81]
Vascular remodelling	ERK1/2, p38, JNK	10% CO_2_ for 1–3 weeks	AngII- and thrombin-induced cell proliferation, deposition of the collagen/extracellular matrix	[82]	[81, 83, 84]
Thrombosis	ERK1/2, p38, JNK	10% CO_2_, acidosis, higher CO_2_/HCO_3_^−^ ratio	Induction of tissue factor expression and NET formation (bronchoalveolar fluid neutrophil infiltration, NF-κB activation, IL-6 and IL-8 production)	[53, 85–87]	[88–92]
Ischaemia‒reperfusion-induced injury	ERK1/2, p38, JNK	Inhaled CO_2_, CO_2_-enriched water (1–1.2 g/l, 10 min once per day), percutaneous CO_2_, EtCO_2_ measurement	Vascular endothelial growth factor (VEGF) stimulation, NO production, cGMP accumulation, cerebral vasodilation, blood‒brain barrier function, haem oxygenase-1 (HO-1) antioxidant activity, attenuation of tissue nitration, inflammation (IL-1β, IL-6 and TNF-α production) and apoptosis	[19, 23, 34, 37, 93–96]	[97–100]
Insulin resistance	ERK1/2, p38, JNK	Incubation of adipocytes in 10% CO_2_	IRS-1 phosphorylation	[21]	[101–108]
Obesity	ERK1/2, p38, JNK	Subcutaneous injections of CO_2_, bathing in neutral bicarbonate ion water	Regulation of adipogenesis, lipogenesis, thermogenesis and browning of white adipose tissue, modification of mitochondrial function	[109–111]	[104, 105, 112–115]
Allergic reactions	ERK1/2, p38	Noninhaled 100% CO_2_ (flow rate 5–10 ml/s), CO_2_ administered intranasally for 10–30 s	Mast cell induction, i.e., activation of NF-κB and AP-1, regulating the expression of histidine decarboxylase and production of histamine and proinflammatory factors, histamine signalling through H1, H2, H3 and H4 receptors	[116–119]	[120–129]
Production of proinflammatory cytokines	ERK1/2, p38, JNK	2–20% CO_2_ for 1–24 h (macrophages or venous blood samples)	Heat shock factor 1 (HSF1)- and NF-κB-dependent transcriptional activity; cytokine secretion, HO-1 antioxidant activity	[18, 34, 37, 49, 55, 130]	[6–9]
Breathing regulation	ERK1/2	Perfusing spinal cord preparations with artificial cerebrospinal fluid equilibrated with 30% CO_2_; CO_2_ inhalation; elevated PaCO_2_	Na^+^ current, Ca^2+^ and Akt signalling, ATP release, erythropoietin	[131–133]	[134, 135]
Memory	ERK1/2	PaCO_2_ 80–100 mm Hg; postacquisition 10% CO_2_ inhalation; CA activation; CA inhibition; acidification	CA activation; protons as a neurotransmitter; acid-sensing ion channel (ASIC); Na^+^ and Ca^2+^ currents	[136–144]	[145–153]
Sleep and circadian rhythm	ERK1/2	Natural fluctuations in CO_2_ levels	CREB-dependent transcription	[154–158]	[159–161]
Sleep apnoea	ERK1/2, p38, JNK	EtCO_2_ raised by 2–4 mm Hg	Regulation of postsynaptic density 95 (PSD-95) expression	[162]	[163, 164]
Anxiety	ERK1/2	5–35% CO_2_ inhalation	Serotonin and BDNF signalling, CREB-dependent transcription	[165]	[166]
Neurodegeneration/neuroprotection	ERK1/2, p38, JNK	50–100 mm Hg PaCO_2_ (0.5–2 h per day, rats), 20% CO_2_ inhalation for 2 min (mice)	Neuronal apoptosis, improvement of exploratory behaviour and total locomotor activity; downregulation of glutamate after brain injury, Ca^2+^ signalling	[167–169]	[1–5, 170–172]
Longevity, cell survival and proliferation	ERK1/2	2–30% CO_2_ (cultured cells); self-produced hypoxic-hypercapnic environment by mice (~ 7% CO_2_); 5 or 20% CO_2_ (*Blastocladia*)	Protein kinase C (PKC) and serotonin signalling (cultured cells), decrease in metabolic rate, body temperature, and food consumption, accelerated wound healing	[14, 173–177]	[5, 14]
Apoptosis	ERK1/2, p38, JNK	CA activation; CA inhibition; acidification; PaCO_2_ 80–108 mm Hg	Regulating pro-survival and pro-death BCL-2 proteins and mitochondrial function; p21 and Akt signalling pathways, HO-1 antioxidant activity	[34, 37, 55, 130, 169, 178–180]	[4, 181–183]
Mitochondrial function	ERK1/2, p38, JNK	Percutaneous CO_2_ (rodents), 5% CO_2_ inhalation (humans)	Mitochondrial biogenesis, fusion, fission, fragmentation and mitophagy, suppression of cerebral metabolic rate of oxygen	[19, 184]	[181, 185–190]

### Changes in CO_2_ levels in the pathogenesis of COVID-19

**Fig. 5:**
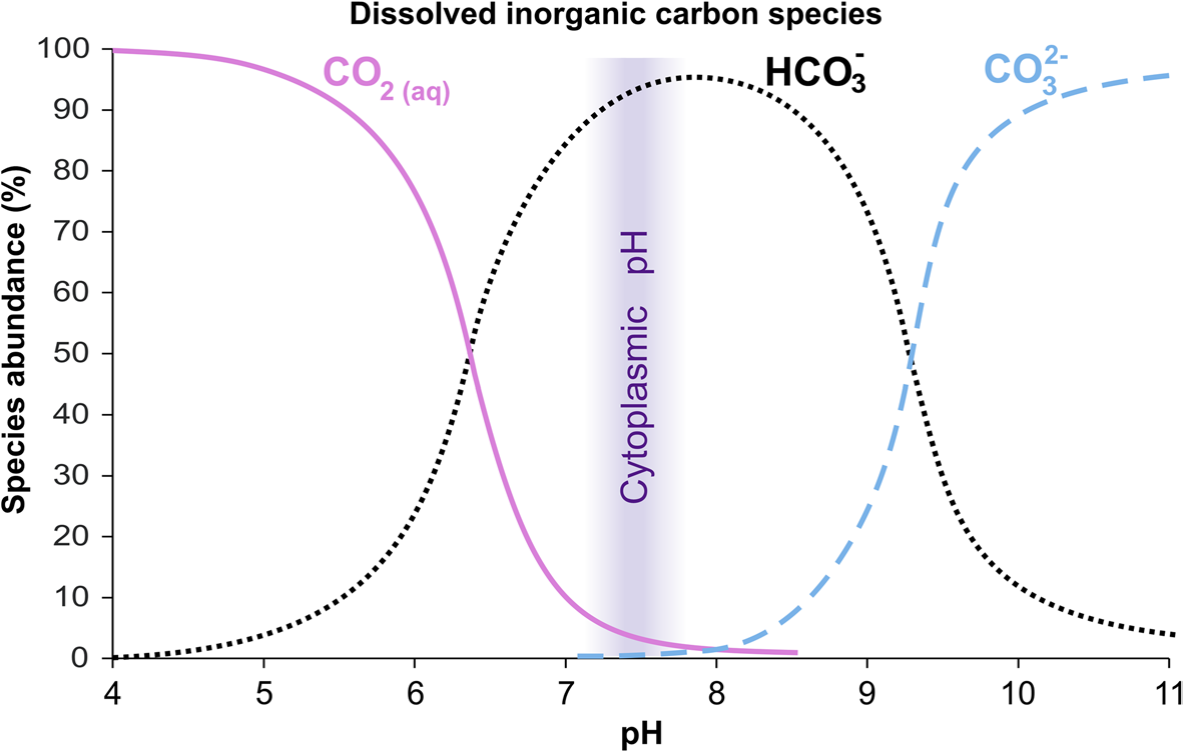
Distribution of the species of dissolved inorganic carbon as a function of change in pH (Bjerrum plot)

CO_2_ is an important component of pH regulatory mechanisms in biological systems. In an aqueous environment, dissolved CO_2_ partially reacts with water to form H_2_CO_3_, which dissociates into HCO_3_^−^ and H^+^. The balance between H_2_CO_3_ and HCO_3_^−^ levels underlies the most important mechanism of maintaining blood pH; H_2_CO_3_ and HCO_3_^−^ are able to neutralize excess bases and acids, respectively. Additionally, the bicarbonate buffer is highly efficient because the respiratory system efficiently removes CO_2_ and dissolved inorganic carbon species are distributed on the basis of pH (Bjerrum plot, Fig. 5). Namely, increasing CO_2_ levels leads to acidification and a shift in the CO_2_/HCO_3_^−^ equilibrium towards a further increase in CO_2_ levels and a decrease in the concentration of HCO_3_^−^. These changes result an increased rate of CO_2_ removal via the lungs and restoration of baseline pH and CO_2_/HCO_3_^−^ levels. The transitions between CO_2_ and HCO_3_^−^ are catalysed by carbonic anhydrases (CAs), which play important roles in the regulation of pH and CO_2_ levels [191]. CAs, depending on the isoform, conditions, pH, and CO_2_/HCO_3_^−^ ratio, may accelerate the hydration of CO_2_ or catalyse the reaction in the opposite direction, thereby affecting the flow of CO_2_ across cell membranes because CO_2_ can be efficiently transported through membranes via diffusion, while HCO_3_^−^ transport through membranes requires the action of transporters [192, 193].

Numerous reports indicate that both HCO_3_^−^ [194–196] and total CO_2_ concentrations are lower in patients who die with COVID-19 than in patients who have recovered from COVID-19 [197–199]. HCO_3_^−^ levels lower than 22 mM have been identified as an important risk factor for mechanical ventilation [200] and a predictor of clinical deterioration in patients with nonsevere COVID-19 [201]. The decrease in the total pool of CO_2_/HCO_3_^−^ in COVID-19 patients is accompanied by an increased level of lactate. The decrease in CO_2_/HCO_3_^−^, which may result from the intensive removal of CO_2_ by the lungs during hyperventilation due to a decrease in blood oxygen saturation (SpO_2_), leads to an increase in the pH of bodily fluids. The increased production of lactate compensates for failure to maintain normal pH. However, the primary cause of the increase in lactate may be an increase in the local glycolysis rate, which is a typical response to infection, inflammation and a decrease in oxygen supply [202]. A reduced rate of aerobic respiration in conjunction with increased glycolytic lactate production leads not only to a decrease in mitochondrial CO_2_ production but also to a decrease in pH. This acidification forces a shift in the CO_2_/HCO_3_^−^ equilibrium, which increases the arterial partial pressure of CO_2_ (PaCO_2_), and elevated PaCO_2_ accelerates the removal of CO_2_ via the lungs, resulting in a reduction in the total pool of CO_2_/HCO_3_^−^.

Since lactate, in contrast to CO_2_, cannot be removed easily via gas exchange, an increase in lactate can lead to a permanent decrease in pH and metabolic acidosis. Although metabolic acidosis does not typically occur in the course of acute COVID-19, blood lactate levels were the highest in nonsurvivors and were higher in hospitalized COVID-19 patients than in ambulatory patients [199, 203, 204]. The greatest differences between a group of patients with COVID-19-related acute respiratory distress syndrome (ARDS) who presented with the "hyperinflammatory" phenotype, with a significantly higher mortality rate and a group of those who presented with the "hypoinflammatory" phenotype, was elevated lactate levels and decreased HCO_3_^−^ levels in patients with the "hyperinflammatory" phenotype; the differences in other markers of inflammation were much less pronounced [205].

Similar to the effect of lactate, permissive and therapeutic hypercapnia leads to a decrease in pH due to an increase in PaCO_2_ resulting from insufficient CO_2_ removal through the lungs and the inhalation of CO_2_, respectively. However, hypercapnia-induced respiratory acidosis does not lead to the many complications attributed to metabolic acidosis and exerts a protective effect in patients with one of many other medical conditions [206–209].

In addition to studies demonstrating the ability of elevated CO_2_ to inhibit the proinflammatory response induced by SARS-CoV-2 elements [10], there are also reports based on randomized trials and case studies showing the benefits of using HCO_3_^−^ in experimental COVID-19 therapies to improve prognosis. Patients with mild COVID-19 who received 14-day nasal NaHCO_3_ irrigation twice daily showed an eightfold lower risk of hospitalization than the overall population [210]. Supplementing standard COVID-19 therapy with 8.4% NaHCO_3_ steam inhalation led to an improvement in clinical parameters in patients with mild to moderate symptoms [211]. A positive effect of 10 ml administration of 4.2% NaHCO_3_ every 6 h was found on mechanically ventilated patients [212].

### Opposing effects of elevated CO_2_ levels

Although the physiological importance of CO_2_ is well understood, its effects at the molecular and cellular levels are poorly understood, and the broad spectrum of CO_2_ concentrations has almost never been compared in CO_2_ signalling studies or for the therapeutic application of CO_2_. The different effects of specific CO_2_ concentrations have been reflected in numerous seemingly contradictory results from research groups that reported results based on different CO_2_ concentrations. Notably, the long-term effect of severe hypercapnia exerted the exact opposite effect of short-term CO_2_ application, e.g., airway muscles were constricted after long-term (3 or 7 days of 10% CO_2_ inhalation) treatment with CO_2_ [213] and were dilated after short-term exposure to elevated CO_2_ [67]. CO_2_ dilates airways that are constricted, e.g., by drugs such as serotonin, methacholine, bethanechol and carbachol, or by the occlusion of the pulmonary artery [68, 69]. Importantly, short- but not the long-term effects of hypercapnia are opposite those of hypocapnia, as transient hypercapnia dilates and hypocapnia constricts airways, as found in dog [70], porcine [71] and rat [72] models. In contrast to acute long-term hypercapnia, slightly elevated CO_2_ levels exert a bronchodilator effect in healthy subjects and in patients with asthma before and after exercise [73, 74]. Consistent with the proposed inhibition of active ERK1/2 induced by elevated CO_2_, inhibition of ERK1/2 increased airway conductance in patients with asthma [75].

The abovementioned opposing effects of CO_2_ at the physiological level are analogous to the opposite effects of CO_2_ on ERK1/2 functions (the activation of inactive ERK1/2 and inhibition of activated ERK1/2). For example, in healthy subjects, elevated PaCO_2_ levels increased pulmonary artery pressure [214], but inhaled 5% CO_2_ reduced preexisting pulmonary artery hypertension (PAH) [77]. Importantly, PAH has been associated with high levels of ERK1/2 and p38 activity [80]. A direct comparison indicated that cyclic stretch–induced injury in human bronchial and alveolar epithelial cells was more efficiently inhibited by hypercapnia applied after cell stretching had begun than by preconditioning the cells via induced hypercapnic acidosis [53]. The specific effects of CO_2_ on MAPK activity and physiology are limited to a relatively narrow range of CO_2_ concentrations. For example, drastically elevated CO_2_ (20%, PaCO_2_ 140 mm Hg) did not inhibit ERK1/2 activity [18] unlike lower CO_2_ levels. Similarly, moderate hypercapnia (PaCO_2_ of 80–100 mm Hg), conferred better protection from high-pressure ventilation–induced inflammatory injury on rat lungs than PaCO_2_ > 100 mm Hg [54].

In summary, prolonged exposure to very high levels of CO_2_ exerts detrimental effects on organisms. However, in the following sections of this review, we focus on the beneficial effects of CO_2_ and its potential therapeutic use, specifically, the short-term effect of slightly elevated CO_2_ concentrations.

### The role of MAPKs in viral infections

MAPKs are activated by viral infection. For example, p38 is activated by hepatitis B virus (HBV), hepatitis C virus (HCV), influenza virus, enterovirus 71, human immunodeficiency virus (HIV) and dengue virus infection [215, 216]. Moreover, MAPKs are involved in many viral infection mechanisms. In addition to induction of a proinflammatory response and regulation of the activity of various types of immune cells during viral infections (e.g., the regulation of CD8^+^ T-cell apoptosis) [217], ERK, JNK and p38 isoforms have been shown to directly support viral multiplication. First, ERK1/2 may positively influence the entry of SARS-CoV-2 into host cells [218]. Second, the phosphorylation of different host proteins by MAPKs facilitates the replication and translation of many viral proteins [219]. Third, efficient nuclear export of viral ribonucleoprotein complexes depends on the activity of ERK1/2 (e.g., influenza virus ribonucleoproteins [220]), and fourth, phosphorylation of viral proteins by MAPKs facilitates viral complex assembly (e.g., p38α phosphorylates the HCV core protein, leading to its oligomerization [215]). Consist with these findings, p38 and ERK1/2 inhibitors impaired the replication of influenza virus and coronaviruses [221–223], including SARS-CoV-2 pseudoviruses, in an in vitro model [215].

### MAPKs in the pathogenesis of COVID-19

Angiotensin-converting enzyme 2 (ACE2), the host receptor of SARS-CoV-2, is a negative regulator of MAPK signalling and thus efficiently prevents both the activation of MAPKs and pneumonia caused by exposure to lipopolysaccharide (LPS) [50], bleomycin [224], cigarette smoke [225] or particulate matter 2.5 (PM2.5) [226]. However, during SARS-CoV and SARS-CoV-2 infection, when receptor ACE2 is bound by the viral spike protein, ACE2 function is disrupted, leading to the activation of MAPKs, the production of proinflammatory cytokines and the pathogenesis of pneumonia or even ARDS [227–232]. In a model mice, COVID-19-like symptoms, including acute lung injury, were caused by inactive SARS-CoV-2 [233] or SARS-CoV-2 spike protein alone [234]. Moreover, ERK1/2 were activated by both SARS-CoV-2 and the spike protein alone in, e.g., human bronchial epithelial cells [10], human dendritic cells [235] and murine primary macrophages [236]. Activation of p38 by spike was found in Vero E6 cells [237], human peripheral blood mononuclear cells [238], HEK293T cells, BHK21 cells [215], murine alveolar macrophages [239] and microglia [240].

Multiple mechanisms lead to the activation of MAPKs by SARS-CoV-2. One such mechanism is renin-angiotensin system (RAS) dysregulation, as SARS-CoV-2 causes internalization of ACE2 by inhibiting the primary function of ACE2, which is the cleavage of angiotensin (Ang) II to form Ang1-7. As a result, the production of Ang1-7 decreases, and the level of AngII increases, leading to the activation of AngII receptor type 1 (AT1) and downstream MAPKs. In addition to ACE2, other membrane receptors have been shown to interact with SARS-CoV-2 and trigger MAPK-mediated signalling; for example, spike protein activates p38 and ERK1/2 via the receptor CD147 in vivo [241] and in primary human cardiac pericytes [242], respectively. The role of MAPKs in SARS-CoV-2 signalling is multifaceted, as the activation of ERK1/2, JNKs and p38 MAPKs is also triggered by the SARS-CoV-2 nucleocapsid protein [243].

The importance of MAPKs has been confirmed not only in classic signalling studies involving MAPK inhibitors but also in many more-objective high-throughput analyses. Proteomic approaches clearly indicated that MAPKs have been found to be among the most highly activated proteins after SARS-CoV-2 infection, regardless of the experimental approaches, cell and sample types evaluated, or period of SARS-CoV-2 infection [237, 244–246]. RNA-seq data revealed that in addition to the regulation of MAPKs by SARS-CoV-2 at the transcriptional level in many cell types [247], the components of the MAPK signalling pathway were also strongly regulated via alternative polyadenylation sites in human peripheral blood mononuclear cells from COVID-19 patients [248].

All types of ACE2-positive immune cells, which are crucial for the pathogenesis of severe COVID-19, can be directly infected by SARS-CoV-2, and as a result of infection, activated MAPK signalling stimulates transcription factors such as NF-κB and AP-1, which trigger the production of proinflammatory cytokines. Despite disputes over whether endothelial cells can be infected by SARS-CoV-2, endothelial cells undoubtedly produce a strong proinflammatory response via MAPKs in patients with severe COVID-19 [249–252]. Vascular endothelial cells in infected organs recruit monocytes/macrophages and neutrophils to inflammation sites and promote further production of proinflammatory cytokines, leading to a cytokine storm [253, 254]. Uncontrolled activation of macrophages not only leads to the secretion of high levels of IFN‐γ, IP-10, IL-6, IL-17, IL-10/23 and TNF-α but also causes a loss of inflammatory coordination mediated by type-I interferons, which is a hallmark of COVID-19. Importantly, type-I interferon production is inhibited by activated ERK1/2 in macrophages [255]. In addition, MAPKs contribute to a decrease in lymphocyte counts, including lymphocyte necrosis and NK and T-cell exhaustion promoted by IL-6, which is commonly observed in COVID-19 patients [254].

In addition to regulating transcription factors, MAPKs regulate other types of effector proteins. For example, p38 and ERK1/2 phosphorylate and thereby increase the catalytic activity of a disintegrin and metalloprotease 17 (ADAM17). ADAM17, due to its proteolytic activity, can release the ectodomains of a variety of proteins; ADAM17 induces ACE2 shedding and the activation of proinflammatory cytokines and fibrotic factors, leading to enhanced organ dysfunction via increased inflammation and fibrosis [256]. The roles of MAPKs in key processes in the pathogenesis of COVID-19 are very broad, as cellular responses to cytokines leading to severe disease in COVID-19 patients depend on MAPK signalling pathways. More detailed information on MAPK signalling in relation to SARS-CoV-2 infection can be found in recent comprehensive reviews [218, 254, 257].

### Hypertension in COVID-19

Hypertension is one of the most common comorbidities that worsens the prognosis of COVID-19. The RAS plays a unique role in regulating blood pressure in patients with COVID-19 due to the direct effect of SARS-CoV-2 on ACE2. SARS-CoV-2 enhances the vasoconstrictive effect of AngII while reducing the amount of Ang1-7, which exert a vasodilating effect. MAPKs constitute a hub for these opposing activities, as both AngII and Ang1-7 signalling is mediated by MAPKs. p38 and ERK1/2 are activated in response to AngII binding by AT1 receptors and induce severe vasoconstriction, increasing blood pressure and heart rate. Moreover, MAPKs have been proposed to be sensors of pressure overload because activation of JNK, p38 and ERK1/2 is proportional to the amount of pressure overload and pressure overload-induced myocardial remodelling in hypertensive patients [258]. In contrast to that of AngII, the activation of Ang1-7 signalling by Ang1-7 binding to the Mas1 receptor leads to inactivation of ERK1/2 via the induction of MAPK phosphatase-1 (MKP-1) in endothelial and vascular smooth muscle cells, which causes not only hypotension but also antiproliferative, antithrombotic and fibrotic effects [259–261].

p38 is overactive in the endothelium and adventitia of hypertensive model rodents in contrast to its activation level in normotensive animals. Activation of p38 in response to AngII activity was transient in normotensive rats but sustained in hypertensive rats [262]. Progressive and sustained hypertension induced by AngII, a high-salt and high-fat diet, or monocrotaline treatment in model mice and rats was reversed by p38 inhibitors; similarly, endothelial dysfunction, vascular cell proliferation, cardiac hypertrophy, and enhanced extracellular matrix and collagen deposition leading to vascular remodelling were reversed [81, 83]. Inhibition of overactivated p38 thus prolonged survival and increased endothelium-dependent vascular relaxation [263].

ERK1/2 are activated in vascular smooth muscle cells, arteries and serum of hypertensive patients. ERK1/2 are crucial for AngII- and thrombin-induced smooth muscle cell proliferation and vascular remodelling, leading to hypertension, atherosclerosis, and accelerated cardiovascular damage [264, 265]. Similar to synthetic MAPK inhibitors, aerobic exercise exerts beneficial effects on vascular and endothelial functions, including the inhibition of vascular smooth muscle remodelling, which led to the acquisition of a hypertensive phenotype by promoting the inactivation of overactive p38 and ERK1/2 in spontaneously hypertensive rats [266]. However, basal ERK1/2 activity has been shown to be essential for maintaining endothelial integrity in vivo, and ERK1/2 loss leads to rapid development of hypertension and death within 5 weeks due to widespread endothelial-to-mesenchymal transition and degradation of endothelial cells in various organs [267].

One of the arguments for the use of CO_2_ in COVID-19 therapy is that the effect of CO_2_ on the circulatory system is consistent with that of Ang1-7; increased CO_2_ causes a decrease in vascular resistance and an increase in blood flow to organs [268, 269]. Experimental COVID-19 therapies based on various vasodilators have improved the prognosis. For example, sildenafil shortened the length of hospital stays and reduced the need for invasive mechanical ventilation [270].

In recent years, research on the effects of CO_2_ on blood pressure has focused on the relationship between CO_2_ levels and PAH, as end-tidal CO_2_ (EtCO_2_) is lower in patients with PAH than in control subjects, and PAH is associated with chronic alveolar hyperventilation. It has been shown that lower EtCO_2_ or PaCO_2_ results in shorter survival in patients with PAH [78, 79].

### Thrombosis

Inflammation of blood vessels in the lungs, heart, brain and other organs is the cause of the most serious complications of severe COVID-19, and COVID-19 is considered a form of inflammatory endotheliitis [249–252]. Typical pathological changes include thrombosis, which may result from inflammation associated with the induction of tissue factor expression (i.e., factors whose mere presence triggers the production of blood clots). In cells that are in contact with platelets, including both monocytes and endothelial cells, the induction of tissue factor depends on p38. Accordingly, MAPK expression has been associated with platelet activation and thrombosis [88, 89]. However, the frequent incidence of thrombosis and the largeness of the thrombi in vessels of COVID-19 patients are largely due to neutrophil activity not inflammatory processes in endothelial cells. In response to SARS-CoV-2, nucleocapsid or spike proteins, IL-6 or, particularly, IL-8, neutrophils extrude neutrophil extracellular traps (NETs), a web of chromatin-based cytoplasmic materials enriched with antimicrobial agents. NETs promote the accumulation of activated platelets and coagulation factors, forming thrombi [90, 271–273].

More NETs have been found in deceased COVID-19 patients than in survivors, and there is a correlation between plasma NET levels and COVID-19 severity [91]. Notably, various studies have consistently shown that the greatest increase in NET production in COVID-19 patients occurs soon after admission to the intensive care unit (ICU). Independent research groups have reported that increased inspiratory airflow and mechanical cell stretch–induced MAPK and NF-κB activation in alveolar macrophages trigger the release of IL-8 and IL-6, which are crucial for NET induction [53, 91, 274].

Treatment with JNK, ERK1/2 or p38 inhibitors abrogates NET formation [90–92]. Similarly, elevated CO_2_ levels inhibit the processes that contribute to the formation of NETs, including bronchoalveolar fluid neutrophil infiltration, NF-κB activation, and the production of IL-6 and IL-8 [53, 85]. Moreover, the effect of CO_2_ on the generation of NETs has been recognized. The formation of NETs is highly dependent on pH and the CO_2_/HCO_3_^−^ ratio; specifically, NET production is induced by high pH and a low HCO_3_^−^ level and inhibited by a high CO_2_ level and low pH [86].

Assuming that the inhibition of MAPKs by CO_2_ is a universal process, the antithrombotic role of CO_2_ may be even broader than previously recognized, as thrombosis in patients with acute COVID-19 results from the binding of activated platelets to NETs, and MAPKs have been well documented in platelet activation in response to both SARS-CoV-2 and the spike protein [275]. In addition, the role of CO_2_ in reducing ROS production, as shown in peripheral occlusive arterial disease, points to a universal antithrombosis-inducing effect of CO_2_ [57]. Accordingly, acidosis promotes a reversible decrease in blood clotting [87].

### Obesity and insulin resistance

Hyperinsulinaemia and hyperglycaemia induce an increase in the SARS-CoV-2 load [276], and high glucose levels lead to higher cytokine and ROS production [277] and activation of ERK1/2 in response to the spike protein in endothelial cells [10]. MAPK-dependent modification of blood vessel development programs, including regulation of the expression of intercellular adhesion molecule-1 (ICAM-1), which is responsible for the recruitment of leukocytes to sites of inflammation [278], increases the risk of patients with type 2 diabetes, among others with vascular disease, experiencing more severe COVID-19 [279]. The incidence of cardiovascular complications, including stroke, and death resulting from venous thromboembolism and pulmonary embolism is several fold higher in diabetic patients than in the general population [280, 281].

MAPKs are involved in various mechanisms leading to obesity, insulin resistance and diabetes, including low-level systemic inflammation; ERK1/2, JNK and p38, which are activated by a high-fat diet, promote the infiltration of monocytes/macrophages into adipose tissue, proinflammatory signalling and dysregulation of immune responses [112, 113]. However, the central role of JNK in the core insulin signalling pathway is the mechanism best understood thus far. Activated JNK (activated, e.g., by hyperglycaemia, free fatty acids, cytokines or ER stress [101–105]) and ERK1/2 phosphorylate insulin receptor substrate-1 (IRS-1) and thus prevent signal transduction after insulin binding to the insulin receptor, leading to alterations in insulin action [106]. The JNK regulation of metabolism is multidimensional. JNK1 knockout protected mice from IRS-1 serine phosphorylation, insulin resistance, fatty liver and diabetes [107, 108]. JNK isoforms are essential regulators in the transition between obesity and type-2 diabetes [104]; JNK1/2 promotes the development of insulin resistance and obesity, whereas JNK3 protects against excessive adiposity [282]. JNK1/2 and p38 promote adipogenesis [283] and regulate lipogenesis, thermogenesis and the browning of white adipose tissue. One of the most important mechanisms among these processes is the modification of mitochondrial function by inhibition of the transcription factor peroxisome proliferator-activated receptor α (PPARα) and uncoupling protein 1 (UCP1) expression in response to, for example, ER stress or a high-fat diet [104, 105, 114, 115].

Direct relationships between insulin resistance, ERK1/2 and p38 activity, and elevated CO_2_ levels have been found in adipocytes, which become insulin resistant as a result of postsurgical trauma. ERK1/2 and p38 are highly activated after surgery. Overnight incubation of adipocytes in 10% CO_2_ inactivated ERK1/2 and p38 and restored insulin receptor and IRS-1 sensitivity to insulin [21]. Consistent with CO_2_-dependent MAPK regulation, subcutaneous injections of CO_2_ reduced body fat [109]. In addition to the use of transcutaneous CO_2_ as carboxytherapy in aesthetic medicine [110], the transcutaneous application of CO_2_ is an efficient treatment for chronic diabetic wounds [284]. Importantly, the major obstacle to wound healing is excessive neutrophil apoptosis caused by the production of NETs [285]; therefore, the beneficial effects of CO_2_ on diabetic wound healing are consistent with the anti-NETosis effect of CO_2_, acidification and MAPK inhibitors [86, 90–92].

### Stroke and ischeamia

Multiple organ failure is the leading cause of COVID-19 mortality. It is a result of, among other causes, organ hypoxia, including hypoxia due to ischaemic stroke, which is a frequent complication of COVID-19. One of the changes observed in the brain in acute stroke patients is a profound reduction in CO_2_ level [286].

In addition to ischaemia, further organ damage is caused by the restoration of blood flow to organs; this damage is known as ischaemia–reperfusion injury. MAPKs in the brain are activated both in response to hypoxia and several minutes after reperfusion. MAPK inhibition has been shown to ameliorate brain injury due to reduced proinflammatory signalling and cell death. Decreasing MAPK activity enhances myelin regeneration, increases blood‒brain barrier function, and suppresses inflammation [97]. Many different drugs that exert neuroprotective effects during cerebral ischaemia inhibit MAPKs and downstream responses, including impairment of IL-1β, IL-6 and TNF-α production by purpurin and the inhibition of apoptosis by tetramethyl pyrazine mediated via JNK inactivation [98–100]. Therefore, blocking the proinflammatory response by inhibiting NF-κB-dependent gene transcription in neurons leads to a reduction in the number of damaged neurons and even to their recovery, reducing the infarct area and death rate of neurons [287].

Therapeutic hypercapnia exerts broadly understood protective effects against reperfusion and oxidative brain injury after ischaemic stroke [136, 286, 288, 289]. In addition to cerebral vasodilation, therapeutic CO_2_ reduces blood‒brain barrier damage [289] and increases sensorimotor activity and spatial memory after focal cerebral ischaemia–reperfusion [136]. In hypoxic regions, pH is lowered to be as low as 6.0–6.5, which confers neuroprotection [206, 209, 290]. An increase in the concentration of protons may result in energy benefits; i.e., an increase in the number of protons contributes to maintenance of a higher membrane potential in the mitochondria, which leads to more efficient ATP production under conditions of oxygen deficiency. This protection has been demonstrated in fish [291], neurons [292] and neuroendocrine prostate cancer cells, in which acidic pH shifted cellular metabolism towards oxidative phosphorylation [293]. This phenomenon may explain the protective role of CO_2_ under conditions of hypoxia and the reduction in oxygen consumption by the brain when CO_2_ levels are increased [184].

Studies on animal models of ischaemic injury have shown that hypercapnic acidosis exerts a protective effect not only on the central nervous system but also on the lung, myocardium, intestine and liver [34, 93–95]. For example, an EtCO_2_ higher than 20 mm Hg at intubation and its increase after resuscitation reduces neurological damage in patients after cardiac arrest [96].

There are known direct relationships between CO_2_ and MAPKs in protection against ischaemia‒reperfusion-induced injury; for example, the protective role of elevated CO_2_ levels in the context of ischaemia‒reperfusion-induced retinal injury is mediated via the inhibition of activated p38 [23]. Hypoxia-induced ERK1/2 activity in mice was suppressed by 10 min percutaneous administration of CO_2_ once per day, which was accompanied a CO_2_-induced increase in ischaemic blood flow and capillary density [19].

### MAPKs and CO_2_ levels in response to mechanical ventilation

Permissive hypercapnia is included in the treatment guidelines for intubated COVID-19 patients in most countries and institutions worldwide. A comparison of hypercapnic and normocapnic COVID-19 patients (PaCO_2_ of 47.1 vs. 39.7 mm Hg, respectively) showed no difference in mortality despite worsened health in the hypercapnic patients; i.e., these patients had a higher body mass index (BMI) and higher number of venous thromboembolic events, chronic obstructive pulmonary disease (COPD) or ARDS [294].

In addition to the effect of hypercapnia on patients with COVID-19, there is debate as to whether hypercapnia should be used for intubated patients, as contradictory results have been obtained from different studies [295]. In addition to reports showing the benefits of hypercapnia, there are studies showing reduced survival times for mechanically ventilated patients with severe hypercapnic acidosis [296, 297]. However, a recent meta-analyses indicated that permissive hypercapnia was associated with lower mortality than imposed hypercapnia under protective ventilation conditions [298]. To date, no studies have been conducted in mechanically ventilated patients with a transient increase in CO_2_ levels (that is, a level sufficient to inhibit the excessive activity of MAPKs in cultured cells [10], corresponding to 5% CO_2_ administered for 15–20 min several times a day). It should be emphasized that CO_2_ is a strong inhibitor of both innate and adaptive immune responses, including inhibition of lymphocyte and natural killer cell cytotoxicity, neutrophil and macrophage migration to sites of infection, and the release of proinflammatory cytokines; therefore, the prolonged use of elevated levels of CO_2_ may lead to weakened immune protection against bacterial infections and sepsis [31, 85], which may worsen the outcome for ICU patients.

The cytokine storm induced by infection with SARS-CoV-2 is enhanced by mechanical ventilation in patients with severe COVID-19. High-pressure mechanical cell stretching exacerbates lung injury, by changing cell histology, increasing lung infiltration with neutrophils, and inducing AEC apoptosis associated with caspase-3 activation [24]. MAPKs are involved in the mechanisms leading to all of these adverse effects in lungs; the most widely recognized of these mechanisms is the role of ERK1/2, which lead to the downstream activation of NF-κB [53], ICAM-1 [54] or ADAM17 [20]. Activation of these signalling pathways as well as ventilation-induced lung injury, AEC apoptosis and increased neutrophil infiltration can be reduced by either elevating the CO_2_ level or inhibiting MAPK function [20, 24, 53, 54, 56].

Since high levels of oxygen support are used in mechanically ventilated patients with severe COVID-19, it seems that there may be additional benefits from the use of elevated CO_2_ levels in these patients, as hyperoxia induces profound lung injury, AEC apoptosis, and ROS and proinflammatory cytokine production [59–66, 299, 300], and CO_2_ inhibits the production of ROS and stimulates the production of antioxidants [57, 58].

### MAPKs and CO_2_ regulate the resorption of alveolar fluid

Symptoms such as shortness of breath, a low SpO_2_ level and lung failure, as well as organ failure, are largely due to oedema fluid (alveolar lining fluid, ALF) flooding alveolar spaces. Excess ALF markedly reduces the amount of oxygen delivered to erythrocytes. In healthy lungs, membrane transporters trigger vectorial ion transport, followed by osmotic influx water, from the apical surface to the basolateral surface of alveolar epithelial cells (AECs), i.e., from the lumina of the alveoli into the lung interstitium and endothelium. Failure of alveolar fluid clearance (AFC) results from a decrease in the level of membrane transporters needed for the flow of ions. AFC is lowered under hypoxic conditions [301], hyperoxic conditions [302, 303], elevated airway pressure [304], pathogen infection and high levels of proinflammatory cytokines, including IL-1β, IL-8, TNF-α and transforming growth factor β1 (TGF-β1), in ALF [38, 301–304].

The main mechanism underlying AFC is the transport of Na^+^ ions across the apical membranes of AECs via epithelial Na^+^ channels (ENaCs). Activated ERK1/2 in AECs phosphorylate the β and γ subunits of ENaC, leading to enhanced interaction of ENaC with Nedd4, an E3 ubiquitin ligase, and to the endocytosis of the ENaC complex and subsequent downregulation [11, 39–42]. Thus, Na^+^ ions accumulate in ALF, leading to an increase in its pH and volume [305]. In addition, ERK1/2 have been indicated to be upstream regulators in the pathway leading to increased degradation of ENaC due to the phosphorylation of Nedd4 by JNK [11]. Inhibition of ERK1/2 or JNK restores the stability of ENaC in the cell membrane, resulting in an increase in AFC. Moreover, in response to IL-1β, p38 inhibits the activity of the α-ENaC gene promoter and the trafficking of ENaC to the apical membranes of type II AECs (ATII) [38]. After absorption into alveoli, Na^+^ ions are eliminated through the basolateral side of AECs mainly via the action of Na/K-ATPase. Both activated ERK1/2 [12] and JNK [25, 26] inhibit Na/K-ATPase, reducing the AFC rate.

In the studies on ENaC and Na/K-ATPase presented above, elevated CO_2_ levels (60–120 mm Hg vs. 40 mm Hg of the control) inhibited AFC but were applied under control conditions to activate MAPKs [11, 12, 25, 26, 42]. Under control conditions, inactive ERK1/2 were activated by elevated CO_2_ levels; therefore, the reduction in AFC rate induced by an increase in CO_2_ level was expected. However, elevating CO_2_ levels shows potential therapeutic value for use under pathological conditions in which excessive ALF production and AFC impairment are observed as a result of high ERK1/2 activity stimulated by infection or inflammation. As active ERK1/2 are inactivated by a transient increase in CO_2_ concentration [10], the therapeutic transient elevation of CO_2_ in the lungs might lead to an increase in AFC under pathological conditions with elevated MAPK activity. These hypotheses are supported, at least in part, by reports showing that transient (20 min) hypercapnia (10% CO_2_) increased AFC when ALF production induced by forskolin was also increased [35].

Moreover, AFC regulation by MAPKs is mediated by aquaporin channels (AQPs), through which water flows following an osmotic gradient. The expression of a key aquaporin, AQP5, is downregulated by p38 and JNK, e.g., in human SPC-A1 cells [43] or murine lungs [44]. Generally, events or factors that activate ERK1/2, p38 and JNK (e.g., infection or cytokines) lead to a decrease in the expression of membrane ion or water transporters and an increase in pulmonary oedema via the action of MAPKs. Consistently, research on inflammation induced by pre-B-cell colony-enhancing factor (PBEF) has shown that ERK1/2 downregulated the main transporters responsible for AFC, i.e., ENaC, Na/K-ATPase, and AQP1 [45].

The commonly held view is that the beneficial therapeutic effect of NaHCO_3_ is due to elevated pH. However, there should be no long-term changes in pH in the lungs after inhalation of NaHCO_3_, as any elevation in pH should be rapidly neutralized by Na^+^ influx into the cytoplasm of AECs via ENaC and across the basolateral membrane into bodily fluids. Otherwise, the increased Na^+^ concentration would be followed by increased secretion of ALF with all the associated negative consequences. In addition, the pH of ALF is regulated by paracellular HCO_3_^−^ flux across the airway epithelium. At the correct (i.e., slightly acidic) pH of ALF, HCO_3_^−^ is secreted. In contrast, when the ALF pH is increased (e.g., in response to infection or proinflammatory cytokines, and presumably after NaHCO_3_ inhalation), HCO_3_^−^ flow is reversed, limiting pH changes [305–307]. These arguments may support the effect of elevated CO_2_ but not an increase in pH in the alveolar epithelium via the therapeutic use of NaHCO_3_.

Among the most widely used and most effective (though still insufficient) drugs in the treatment of acute COVID-19 is highly concentrated dexamethasone, a corticosteroid that inhibits the activity of MAPKs [308]. In addition to its anti-inflammatory effect, dexamethasone increases the amount of ENaC in AECs by inhibiting ERK1/2 [46, 47] and increases the AFC rate. However, under many pathological conditions where ERK1/2 are activated, decreased sensitivity to glucocorticoids is observed [309, 310]. The molecular mechanism underlying the dexamethasone-dependent regulation of MAPKs is the upregulation of MKP-1 and, as a result, increased inactivation of MAPKs. Transcriptomic data indicate that SARS-CoV-2 infection leads to downregulation of MKP-1, reducing cell sensitivity to corticosteroids [311, 312]. Consequently, in contrast to elevated CO_2_, dexamethasone was not able to block the activity of ERK1/2 induced by the spike protein in bronchial epithelial cells in the presence of IFN-γ and TNF-α [10].

### MAPKs and CO_2_ in allergy

Among the immune cells with functional SARS-CoV-2-entry machinery, i.e., the expression of ACE2 and TMPRSS2, there are mast cells that handle allergic reactions [313]. Mast cells are stimulated during SARS-CoV-2 infection via ERK1/2, which activate the transcription factors NF-κB and AP-1, leading to the release of a wide variety of proinflammatory factors [120–122]. In addition, ERK1/2 stimulate histamine production by regulating the expression of histidine decarboxylase [123, 124]. The histamine signal is received by a wide range of cells in various organs through H1, H2, H3 and H4 receptors, and signalling downstream of each of these receptors is mediated by MAPKs [125–127]. Histamine signalling mediated via ERK1/2 also regulates the activity of the mast cells themselves, regulating the production of important molecules such as nerve growth factor (NGF) following activation of the H1 receptor [122] and IL-6, TNF-α, TGF-β1, IL-8, macrophage inflammatory protein-1α (MIP-1α/CCL3), and monocyte chemoattractant protein-1 (MCP-1/CCL2) in response to H4 receptor stimulation [128].

Because of the participation of mast cells in the course of COVID-19, antihistamines are among the most commonly prescribed medications in COVID-19 therapy. The importance of H1 receptor antagonists has been confirmed not only in numerous in silico and in vitro studies but also through its use in the clinic to alleviate the symptoms of COVID-19 in patients. Information on various H1 receptor modulators in the treatment of COVID-19 can be found in a recent review article [314]. In addition, competitive inhibitors of histamine H2 receptors, such as famotidine, are very effective in relieving mild COVID-19 symptoms [315] and in protecting against death and intubation [124, 316], although the exact molecular mechanisms are yet to be elucidated.

The inhibition of overactive MAPKs by elevated CO_2_ levels in allergic reactions is of particular interest because MAPK/NF-κB-inhibiting drugs (e.g., lidocaine or p38 inhibitors) [129] and elevated CO_2_ levels are both effective in treating allergy symptoms. In various clinical trials, noninhaled 100% CO_2_ (flow rate 5–10 ml/s) was effective in the treatment of allergic rhinitis [116]. The effect of a single dose of CO_2_ administered intranasally for 10–30 s lasted 4 to 6 h, and a 60-s dose lasted 24 h. Similarly, after a 20-s exposure to CO_2_ prior to allergen exposure, the acute responses to allergen challenge were reduced; for example, there were a significant reduction in sneezing, secretion weight and bilateral rhinorrhoea symptoms. CO_2_ also led to inhibited histamine release [117]. Accordingly, CO_2_ inhibited mast cell degranulation and histamine release in vitro [118]. Moreover, a decrease in PaCO_2_ is one of the most common initial symptoms of anaphylactic reactions [119], suggesting the benefits of using elevated CO_2_ levels for inhibiting the most severe allergic reactions. Taken together, the evidence suggests that the regulation of CO_2_-MAPK pathways in the inhibition of allergic reactions is a promising direction for future research.

### COVID-19 and smoking

Early in the COVID-19 pandemic, controversial analyses indicated that, contrary to predictions, smoking did not only not worsen the prognosis but also may have protected patients against the development of severe COVID-19 symptoms [317–319]. Various studies have pointed to a lower rate of daily smokers presenting with symptomatic COVID-19 [319] and a lower risk of hospitalization, serious illness or death compared to the general population [320–326]. Interestingly, even in studies that concluded that smoking worsened the prognosis of COVID-19, the proportion of smokers with severe COVID-19 compared to the proportion of smokers in the general population showed that smoking conferred a protective effect [327].

Although the reasons for the potential protective effects of tobacco smoke are unknown, reports of the beneficial effects of smoking have been increasing, so clinical trials have been launched based on the hypothesis that nicotine plays a protective role against the development of COVID-19. Although the arguments presented in this paper may suggest that the effects of smoking considered to be positive may be due to the inhalation of elevated levels of CO_2_ during smoking; however, this hypothesis should be considered with caution. Notably, as smokers have a much higher risk of cardiovascular and respiratory disease, their milder COVID-19 cases may have be due to the protective effects of the medications they take. On the other hand, numerous comorbidities in this group of patients, compared to the general population, may suggest a very strong protective effect of inhaled tobacco smoke.

### MAPKs and CO_2_ in breathing regulation

Shortness of breath is a typical symptom caused by infection with early variants of SARS-CoV-2; thus, the regulation of breathing plays an important role in the pathogenesis of COVID-19. During wakefulness, CO_2_ levels are sensed mainly by central chemoreceptors, i.e., the chemosensory neurons in the medulla oblongata sensitive to CO_2_, which also sense a decrease in pH of cerebrospinal fluid, and carotid body chemoreceptors determine the sensitivity of central chemoreceptors to CO_2_ [131, 132]. An elevated CO_2_ level is the primary factor for increasing ventilation and blood flow to the brain. However, prolonged hypercapnia reduces the sensitivity of chemoreceptors to CO_2_, leading to slower of CO_2_-induced rapid breathing over time. Similarly, breathing becomes faster as the concentration of inspired CO_2_ increases up to 9–10%, and a further increase in CO_2_ concentration leads to a decrease in ventilation.

The mechanics of breathing regulation, especially, CO_2_ level sensing, are very poorly understood at the molecular level. Mitochondria appear to be crucial for the response to elevated CO_2_, as CO_2_ induces the immediate release of ATP from chemosensitive regions of the ventral surface of the medulla oblongata [133]. ATP release is mediated by connexin 26 [328] and potentiates the release of acetylcholine [329]. Studies with model animals indicated that ERK1/2 were crucial for the regulation of respiration, as inhibition of ERK1/2 in brainstem preparations led to impaired breathing responses to CO_2_-induced acidosis [134].

One of the most important goals in the treatment of severe COVID-19 is an increase in low SpO_2_ levels. Brief inhalation of CO_2_ increases SpO_2_ [184]. The increase in SpO_2_ was immediate, within ~ 1 min, and 2-min sessions of inhaled 4, 8, or 12% CO_2_ with nebulized perflubron, a synthetic surfactant, caused SpO_2_ to increase by 1.7, 1.9 and 2.3 percentage points, respectively. The increase in SpO_2_ was maintained for 20 min, and subsequent inhalation treatments (twice per day) further stabilized the patient, suggesting a cumulative beneficial effect. Statistically significant increases in SpO_2_ in patients with cystic fibrosis are maintained 9 days after completion of the 5-day series of CO_2_ inhalation [330]. In another trial, inhalation of 8% CO_2_ increased SpO_2_ in subjects with mild allergic asthma after allergen-induced bronchoconstriction [331].

In addition to increasing SpO_2_, inhaled CO_2_ increase the supply of oxygen to tissues because CO_2_ allows oxygen to be released from haemoglobin (the Bohr effect) [332–334]. Thus, the CO_2_ inhalation treatment led to simultaneous increases in SpO_2_, better utilization of the oxygen in tissues because of the Bohr effect, and increases in blood flow due to vasodilation, which may support the use of less intense oxygen therapy.

### CO_2_ and sleep

Sleep disturbances are common symptoms of both COVID-19 and post-COVID syndrome. Changes in CO_2_ levels are associated with sleep onset, wakening, and sleep stages. During sleep, PaCO_2_ is typically 2–8 mm Hg higher than it is during waking hours, depending on the sleep stage, and the sensitivity of the medulla oblongata chemosensors to CO_2_ decreases and hypoventilation occurs [154, 155]. Thus, an increase in PaCO_2_ of 2–8 mm Hg in mechanically ventilated patients may not be considered hypercapnic but a desirable baseline physiological level.

The intensity of neuromotor responses regulating breathing is significantly reduced during sleep compared to that during wakefulness; therefore, only marked increase in hypoxemia or hypercapnia increase ventilation during sleep. Similarly, waking up may be triggered only by a decrease in SpO_2_ to 70% or an increase in PaCO_2_ by 15 mm Hg compared to eupnoeic levels. In contrast, the physiological tolerance for decreased PaCO_2_ during sleep is low, since a decrease in PaCO_2_ by 3–6 mm Hg during sleep leads to sleep apnoea. Therefore, eupnoeic PaCO_2_ when awake may not be enough to sustain eupnoeic breathing during sleep [156, 157].

Maintaining waking PaCO_2_ leads to long-term sleep deprivation in patients in a medically induced coma. In mechanically ventilated patients, REM sleep is absent (and markedly reduced in patients with noninvasive mechanical ventilation) [335–337]. REM sleep is associated with an additional increase in PaCO_2_ of 1–2 mm Hg [158], local increases in low-frequency oscillations and global decreases in high-frequency oscillations in the electroencephalography (EEG) spectrum [338]. CO_2_ is the determining factor for changes in brain activity; inhaled CO_2_ leads to an increase in low-frequency power in the EEG spectrum [184]. There is a close connection between ERK1/2 and CO_2_ in the regulation of REM sleep; active ERK1/2-brain-derived neurotrophic factor (BDNF) signalling in the pedunculopontine tegmentum promotes homeostatic control of REM sleep [339]. Neurotransmitters involved in the regulation of sleep, the circadian rhythm and treatments that prevent major depressive disorder activate the key transcription factor cAMP response element-binding (CREB) via ERK1/2 signalling [159–161].

In mechanically ventilated patients, CO_2_ supplementation is particularly beneficial because mechanical ventilation decreases both pO_2_ and PaCO_2_ in the lungs. Inhalation of 1.5–2% CO_2_ is required to maintain the target EtCO_2_ of 4.7–4.9% in mechanically ventilated patients [340]. Interestingly, studies in model animals have indicated that the decrease in pO_2_ and PaCO_2_ that occurs in mechanically ventilated lungs can be inhibited by ERK1/2, p38 and JNK inhibitors [341].

### Regulation of memory by MAPKs, CO_2_ and mitochondria

The levels of cellular CO_2_ produced via aerobic oxidation of carbohydrates are higher than those produced via other ATP synthesis pathways. Therefore, CO_2_ signalling particularly affects organs that consume carbohydrates as their main sources of energy. Therefore, CO_2_ is an important regulator of brain function, and as much as 20% of all CO_2_ in the body is generated in the brain, even though the brain represents approximately 2% of human body weight. Hypocapnia occurs in 74% of individuals post-COVID, and patients show neurological symptoms: fatigue, insomnia, depression and post-COVID brain fog [342]. Hypocapnia triggers known negative effects in various neurological diseases and conditions, such as severe traumatic brain injury, and ischaemic or haemorrhagic strokes. Moreover, hypocapnia negatively affects neonatal brain development, and the harmful effects can be reversed by inhaling 5% CO_2_ [343]. CO_2_ inhalation enhances the formation of memories and long-term memory [136–139]. The role of CO_2_ in the regulation of memory has been indirectly discerned from numerous studies on CAs. CA inhibitors (e.g., acetazolamide) impair [140], and administration of CA activators enhances [141, 142] memory and learning in model animals.

The positive effect of activated CAs on memorization and learning is mediated through the activation of ERK1/2 in the cerebral cortex and hippocampus, among other brain structures, and ERK1/2 are key elements required for the formation, retrieval, consolidation, reconsolidation, and persistence of memory [145–147]. Factors that increase cognitive abilities (e.g., amphetamine, methamphetamine, D-phenylalanine, phentermine, mephentermine, chlorphentermine and cocaine- and amphetamine-regulated transcript (CART), and neuropeptide) exert effects via ERK1/2 activation in the hippocampus [148–150] and potently activate CAs [141–144]. In contrast, memory-impairing drugs, such as hypnotic, amnestic and anaesthetic agents (e.g., butylphthalide, ketamine, midazolam, pentobarbital, isoflurane, propofol and scopolamine) reduce ERK1/2 activity in the brain [151, 152]. All naturally occurring mutations in the genes encoding ERK1/2 (*MAPK1* and *MAPK3*) in humans, including mutation in noncoding regions of the genes, lead to cognitive impairment. Moreover, *Erk2*^−/−^ mice showed a deficit in long-term memory [153].

Notably, the connections between ERK1/2 and mitochondria, which are sites of CO_2_ production, are important because endogenous CO_2_ is a natural regulator of neuron function, and mitochondria are critical to neuron function, including memory formation [344]. Local synaptic ATP production must be adjusted to meet high energy demand [345]. Mitochondrial mobility in neurons is essential for the formation of memories, and during learning, the number of mitochondria increases, but the size of mitochondria decreases. These mitochondrial changes promote the formation of multicontact synapses, which increases the information storage capacity of new synapses [346]. In contrast, in the neurons of the ageing brain, mitochondria become elongated as autophagy, fusion and fission of the mitochondria are disrupted [347].

The abovementioned processes are regulated by ERK1/2, which activate mitochondrial fission and inhibit fusion [185]. Cycles of fission and fusion help the mitochondrial network adapt to changing metabolic needs and are part of a fusion–fission–mitophagy quality control pathway enabling the removal of dysfunctional mitochondria. The large GTPase dynamin‐related protein 1 (DRP1) is recruited to sites of mitochondrial constriction, where it forms a higher-order ring structure that promotes fission via GTP‐dependent scission. The phosphorylation of DRP1 at Ser616 by ERK1/2 promotes mitochondrial translocation of DRP1 and subsequent mitochondrial fission/fragmentation [181, 186, 187]. ERK1/2 have been identified in mitochondria in several independent studies and are strongly regulated by essential mitochondrial products, i.e., CO_2_, ATP and H_2_O_2_ [348]. Moreover, ERK1/2 regulate mitochondrial biogenesis; in general, they induce mitochondrial biogenesis under control conditions and inhibit it under pathological conditions [188, 189]. ERK1/2 are involved in the regulation of the transition from mitochondrial respiration to glycolysis [190], and it has been proposed that mitochondrial ERK1/2 provide information about mitochondrial energetic and redox status to the nuclear pathways [349].

Mitochondrial dysfunction is closely associated with a variety of neurological disorders and ultimately leads to neuronal apoptosis. Synaptic mitochondrial dysfunction occurs during ageing and correlates with age-related memory loss. Synaptic mitochondria are the primary targets of both amyloid-β [350, 351] and phosphorylated tau [352] toxicity, which contributes to synaptic and memory impairment in Alzheimer's disease. Restoration of mitochondrial function is being intensively developed as a therapeutic strategy for dementia and learning and memory problems [351, 353, 354].

Understanding the relationship between CO_2_, ERK1/2 and mitochondria may allow for deeper insight into memory mechanisms and the emergence of new therapeutic possibilities. Neurodegenerative disorders are inflammatory in nature and characterized by contradictions in the functioning of ERK1/2; ERK1/2 are essential for the normal function of neurons, but their excessive activity leads to the development of inflammation. Therefore, despite the positive effect of active ERK1/2 on cognition, increased ERK1/2 activity is evident in neurodegenerative disorders. Thus, MAPK inhibition promotes neuroprotection [1, 170–172]. However, complete inhibition of ERK1/2 induced by synthetic inhibitors can be problematic, since basal ERK1/2 activity is essential for neuronal survival and memory. Elevated CO_2_ may be a universal therapeutic alternative that attenuates these drawbacks. CO_2_, on the one hand, may stimulate insufficiently active ERK1/2 (e.g., in an ageing brain) but, on the other hand, inhibit overactive proinflammatory MAPKs.

In addition to the role of mitochondria in the mechanisms underlying memory discussed here, the understanding of the effects of SARS-CoV-2 on mitochondria, including fusion, fission, mitophagy, metabolic reprogramming, and of the regulation of the immune response and apoptosis, has increased, and these effects are thoroughly discussed in numerous recent reviews [355–357].

### MAPKs and CO_2_ in cell survival and apoptosis

Lymphocytes are among the cells whose death contributes most to severe COVID-19, with T-cell apoptosis accounting for T lymphopenia in patients with severe COVID-19 [358]. Negative regulation of apoptosis by ERK1/2 has previously been shown to be required to ensure survival of T and B lymphocytes [182]. ERK1/2 promote cell survival by activating prosurvival BCL-2 proteins (BCL-2, BCL-xL and MCL1) and repressing prodeath protein (BAD, BIM, BMF and PUMA) activity, including the key mechanism underlying the phosphorylation of BIM_EL_ by ERK1/2, thereby preventing homo-oligomerization of BAX, a proapoptotic member of the BCL-2 family responsible for the permeabilization of the mitochondrial outer membrane; loss of potential across the inner mitochondrial membrane and cytochrome c release [181, 183].

In response to a large cellular imbalance, e.g., caused by DNA damage or excessive inflammation, ERK1/2 are hyperactivated and may exert a proapoptotic effect [4, 183]. However, in general, ERK1/2 promote cell survival and proliferation, whereas activation of JNK and p38 may induce apoptosis [359]. Since a slight increase in CO_2_ concentration (from 5 to 8%) activates ERK1/2 and inhibits p38 and JNK under control conditions [10], the question arises: Does such a slight increase in CO_2_ levels support the prosurvival activity of ERK1/2 while inhibiting apoptotic p38 and JNK? Indeed, the overlapping functions of CO_2_ and ERK1/2 include the regulation of apoptosis and longevity, and the lifespan of mammals positively correlates with blood PaCO_2_ and HCO_3_^−^ [5, 167, 173, 174]. Moreover, human cells are unable to proliferate without CO_2_, and elevated CO_2_ levels support cell proliferation [175, 176]; CO_2_ has been shown to exert an effect via ERK1/2 in a cell line derived from human small cell lung cancer [14]. Furthermore, hypercapnia induces the expression of anti-apoptotic BCL-2 and BCL-xL and inhibits autophagy in macrophages [178] and ischaemic penumbra astrocytes and neurons [167]. Similarly, CAs [179] and acidification [360], which shifts the CO_2_/HCO_3_^−^ equilibrium towards an increase in the level of CO_2_, activate ERK1/2 and thus delay neutrophil apoptosis, improving neutrophil migration and wound healing. Therefore, the inhibition of CAIX resulted in a reduction in the level of active ERK1/2 and reduced neutrophil viability and mitochondrial function [180]. An extreme case of the prosurvival effect of CO_2_ involves the accelerated growth of the aquatic uniflagellate phycomycetes *Blastocladia ramosa* and *Blastocladia pringsheimii* induced by an increase in CO_2_ of 5–20% [177].

It is believed that at the time when life was formed, the Earth’s atmosphere and water reservoirs contained much more CO_2_ than they contain today. To survive, primitive organisms needed to be adapted to the natural environment, i.e., to high concentrations of CO_2_. The change to the oxidizing atmosphere was followed by endosymbiosis, allowing eukaryotes to maintain high levels of CO_2_ by producing CO_2_ inside the cells in mitochondria.

Typical mitochondrial respiration involving oxygen consumption is associated with CO_2_ production during the entry of pyruvate into the Krebs cycle and two stages of the Krebs cycle. Anaerobic energy production by organisms or cells consuming organic compounds is also associated with increased CO_2_ levels. However, this production can be mediated by acidification, e.g., by the glycolytic production of lactate, which shifts the CO_2_/HCO_3_^−^ equilibrium towards an increased CO_2_ concentration. There are species of multicellular eukaryotes that tolerate periods of complete oxygen deprivation; e.g., the larvae of oriental fruit flies (*Bactrocera dorsalis*) can tolerate up to 24 h of anoxia without a significant reduction in survival [361]. In several species of fish adapted to life under completely oxygen-deprived conditions; for example, one of the clearest differences in crucian carp compared to aerobic species is the activity of pyruvate decarboxylase, which produces CO_2_ independent of the Krebs cycle [362, 363]. In addition, some turtles survive complete anoxic conditions, and upregulation of the prosurvival proteins ERK1/2 and suppression of p38 and JNK underlie neuronal survival [364, 365].

## Conclusions and future perspectives

**Fig. 6:**
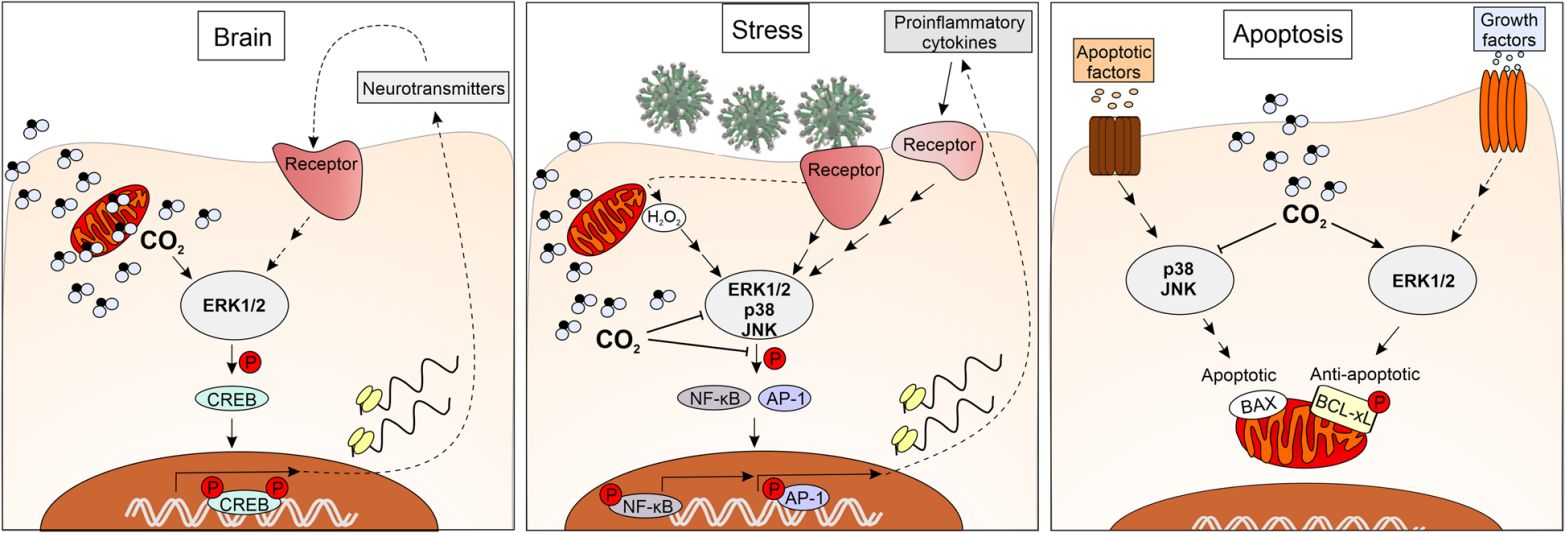
Cellular MAPK signalling pathways regulated by CO_2_. Dashed lines represent secondary or concurrent signalling pathways. Multiple arrows indicate indirect regulation

Considering the previously published data presented here, it can be concluded that MAPKs play a central role in regulating cellular responses to changing CO_2_ levels (Fig. 6). Detailed studies on the regulatory mechanisms MAPK activity by CO_2_ suggest that reassessing the functioning of MAPK signalling pathways while taking into account the level of CO_2_, as each MAPK pathway may function differently under altered CO_2_ levels, is needed. The effect of CO_2_ on the activity of ERK1/2, JNKs and p38 MAPKs varies depending on its concentration. In addition, individual MAPKs function differently in different signalling pathways. Therefore, it can be expected that relatively narrow ranges of CO_2_ concentrations will modify MAPK activity in the desired way under specific conditions. Therefore, the basis for future research and the first priority is to determine the effects of specific CO_2_ concentrations on individual MAPK isoforms in detail; this research should include in vitro studies using recombinant MAPKs, to confirm the direct CO_2_-sensing ability of MAPKs. We expect that these studies will accelerate CO_2_ research because MAPKs are involved in many developmental processes and oncogenesis.

Advances in our understanding of CO_2_ signalling have been relatively slow. The first genome-wide proteomic, transcriptomic [366] and genetic [367] analyses of the yeast CO_2_ response were reported only recently. These data support the hypotheses presented in this paper by showing that the MAPK pathway is critical for CO_2_ sensing and CO_2_ signalling in yeast. Hope for progress in CO_2_ research is offered via the recent development of selective fluorescent CO_2_ molecular sensors, which are expected to lead to breakthrough insights into biochemical processes [368], and detection methods for carboxylation of the amine group in lysine residues [369].

MAPKs are signalling molecules connecting various aspects of the inflammatory response to viral infection, including SARS-CoV-2 infection, and comorbidity pathogenesis. In addition, CO_2_ appears to be a molecule that universally counteracts the MAPK-induced proinflammatory response, including in patients with severe course COVID-19 and complications leading to death. The benefits of using elevated CO_2_ levels in the treatment of various diseases are reflected in numerous completed and ongoing clinical trials established to evaluated CO_2_ use as a medication. Advances in research on the regulation of CO_2_-MAPK may significantly increase the number of new therapeutic applications of CO_2_ because a number of MAPK inhibitors have been approved as potent drugs for the treatment of numerous diseases, including cancers, and a significant number of these inhibitors are under evaluation in different stages of clinical trials. However, many MAPK inhibitors cannot be fully exploited at the most effective concentrations due to their toxicity and resistance mechanisms; for example, the activation of ERK5 can enable cells to bypass RAF-MEK1/2-ERK1/2 inhibitors [370]. CO_2_, which is safe under controlled conditions [331, 371], may overcome these limitations because it inhibits various ERKs, JNKs and p38 MAPKs when administered in a precise concentration range. Other advantages involve the delivery of CO_2_ to cells independent of membrane transporters and the possibility of administering very high concentrations of CO_2_ locally without causing systemic effects and only nominal effects on the cells surrounding the CO_2_ application site.

In conclusion, understanding the molecular mechanisms underlying CO_2_-dependent regulation of MAPKs, including the opposing effects of elevated CO_2_ on active and inactive ERK1/2, is essential to precisely guide the development of therapeutic CO_2_ applications.

## Data Availability

Not applicable.

## Abbreviations

15-HETE: 15-Hydroxyeicosatetraenoic acid
ACE2: Angiotensin-converting enzyme 2
ADAM17: A disintegrin and metalloprotease 17
AEC: Alveolar epithelial cell
AFC: Alveolar fluid clearance
Akap1: A-kinase anchoring protein 1
ALF: Alveolar lining fluid
Ang: Angiotensin
AQP: Aquaporin
ARDS: Acute respiratory distress syndrome
ASIC: Acid-sensing ion channel
AT1: Angiotensin II receptor type 1
ATII cell: Type II alveolar epithelial cell
BDNF: Brain-derived neurotrophic factor
BMI: Body mass index
CA: Carbonic anhydrase
CART: Cocaine- and amphetamine-regulated transcript
COPD: Chronic obstructive pulmonary disease
CREB: cAMP response element-binding
DRP1: Dynamin‐related protein 1
EEG: Electroencephalography
EGFR: Epidermal growth factor receptor
ENaC: Epithelial Na^+^ channel
ER: Endoplasmic reticulum
ERK1/2: Extracellular signal-regulated kinases 1 and 2
EtCO_2_: End-tidal CO_2_
HBV: Hepatitis B virus
HCV: Hepatitis C virus
HIV: Human immunodeficiency virus
HO-1: Heme oxygenase-1
HSF1: Heat shock factor 1
ICAM-1: Intercellular adhesion molecule-1
ICU: Intensive care unit
IFN-γ: Interferon-gamma
IL: Interleukin
IRS-1: Insulin receptor substrate 1
JNK: c-Jun N-terminal kinase
LPS: Lipopolysaccharide
MAPK: Mitogen-activated protein kinase
MCP-1/CCL2: Monocyte chemoattractant protein-1
MIP-1α/CCL3: Macrophage inflammatory protein-1α
MKP-1: MAPK phosphatase 1
NET: Neutrophil extracellular trap
NK: Natural killer
PaCO_2_: Arterial partial pressure of CO_2_
PAH: Pulmonary artery hypertension
PBEF: Pre-B-cell colony enhancing factor
PKC: Protein kinase C
PKA: Protein kinase A
PM2.5: Particulate matter 2.5
PPARα: Peroxisome proliferator-activated receptor α
PSD-95: Postsynaptic density 95
ROS: Reactive oxygen species
SMAD3: Small mothers against decapentaplegic 3
TGF-β1: Transforming growth factor β1
TNF-α: Tumour necrosis factor-α
UCP1: Uncoupling protein 1
VEGF: Vascular endothelial growth factor
